# The impact of the combination of *KIT* mutation and minimal residual disease on outcome in t(8;21) acute myeloid leukemia

**DOI:** 10.1038/s41408-021-00461-z

**Published:** 2021-04-01

**Authors:** Ya-Zhen Qin, Qian Jiang, Yu Wang, Hao Jiang, Lan-Ping Xu, Xiao-Su Zhao, Xiao-Hui Zhang, Kai-Yan Liu, Xiao-Jun Huang

**Affiliations:** Peking University People’s Hospital, Peking University Institute of Hematology, National Clinical Research Center for Hematologic Disease, Beijing Key Laboratory of Hematopoietic Stem Cell Transplantation, Beijing, China

**Keywords:** Risk factors, Acute myeloid leukaemia

Dear Editor,

Acute myeloid leukemia (AML) with t(8;21) is a heterogeneous disease and needs to be further stratified^1–3^. We previously reported that high-risk t(8;21) AML patients benefited from allogeneic hematopoietic stem cell transplantation (allo-HSCT)^4^, which implied that risk stratification could guide appropriate treatment selection for t(8;21) AML.

At present, *KIT* mutation is still the only widely accepted gene mutation with strong prognostic significance in t(8;21) AML^5–10^. Furthermore, *RUNX1-RUNX1T1* transcript levels after treatment has been routinely tested to monitor minimal residual disease (MRD) and established as a powerful marker to predict relapse and guide treatment^4,11–14^. However, report on how to combine *KIT* mutation status with MRD levels to assess prognosis remains absent to date.

The current study included 287 t(8;21) AML patients who consecutively received treatment and achieved complete remission (CR) in our center from February 2009 to December 2019. The median age at diagnosis was 36 (range, 15–65) years. Information about patient treatment and samples availability before 2nd consolidation was shown in Fig. S1. The study was conducted in accordance with the Declaration of Helsinki and was approved by the Ethics Committee of the Peking University People’s Hospital. The cutoff date for the follow-up was October 31, 2020.

As we have previously reported^4^, induction chemotherapy was composed of 1–2 cycles of induction with the “3 + 7” regimen or the HAA regimen (homoharringtonine, cytarabine, and aclarubicin), and the post-remission therapy included intermediate-dose cytarabine-based chemotherapy (IDAC; 1–2 g/m^2^ every 12 h for 3 days; 2–4 cycles of cytarabine followed by 2–4 cycles of the “3 + 7”regimen), autologous-hematopoietic stem cell transplantation (auto-HSCT), or allogeneic-HSCT (allo-HSCT). After achieving CR, 162 patients received chemotherapy alone, 9 received chemotherapy followed by auto-HSCT, and 116 received chemotherapy followed by allo-HSCT (matched sibling donor, *n* = 38; haploidentical related donor, *n* = 72; matched unrelated donor, *n* = 6). The indications for the allo-HSCT were described in our previous studies^14,15^.

270 and 17 patients individually achieved CR after 1–2 and 3–4 cycles of induction, 80 patients (27.9%) experienced hematological relapse, and 250 patients (87.1%) were alive at the last follow-up. The median follow-up time was 28.5 (range, 3.3–109.0) months for the surviving patients. The 3-year cumulative incidence of relapse (CIR) and overall survival (OS) rate were 29.9% [95% confidence interval (CI), 21.6–38.6%] and 85.0% (95% CI, 79.5–89.1%), respectively.

Overall, 120 patients (41.8%) had *KIT* mutations (246 patients were screened at diagnosis and 41 screened after treatment with *RUNX1-RUNX1T1* transcript levels higher than 5%). The mutations were categorized into the following six types: sole D816 (18.5%, *n* = 53; 38 D816V, 8 D816Y and 7 D816H), sole N822 (11.1%, *n* = 32; all were N822K), sole D820 (2.4%, *n* = 7; 5 D820G, 1 D820A, 1 D820Y), sole R815_D816delins (1.4%, *n* = 4), sole exon 8 delins (4.2%, *n* = 12, abbreviated as e8 thereafter) and compound mutations (4.2%, *n* = 12). The types of compound mutations were as follows: 5 D816 + D816, 1 D816 + I817, 1 D816 + D820, 1 D816 + N822, 2 D816 + e8, 1 D820 + N822 and 1 D820 + e8. Thus, all of the compound mutations contained D816 or/and D820 mutations.

First, patients were grouped according to their *KIT* mutation status. In our previous study, t(8;21) AML patients were categorized into D816/D820 mutation and N822/e8/WT groups and D816/D820 mutation was demonstrated to be an independent adverse prognostic factor for both relapse free survival and OS^10^. Here, patients with D816, compound mutations, 815_816delins and D820 mutations had similar 3-year CIR in the whole cohort and if censoring at the time of allo-HSCT [62.1% (95% CI, 48.1–73.3%) vs. 58.3% (95% CI, 31.4–77.8%) vs. 50.0% (95% CI, 5.8–84.5%) vs. 57.1% (95% CI, 5.0–90.0%), *P* = 0.60; censoring: *P* = 0.44, Fig. S2]. Because all patients in the above four groups had D816 or/and D820 mutations, they were merged and defined as *KIT*^D816/D820^ (*n* = 76). In addition, because patients with e8 mutations had similar 3-year CIR to those with no mutation [8.3% (95% CI, 0–70.5%) vs. 19.9% (95% CI, 9.2–33.6%), *P* = 0.27; censoring: *P* = 0.42, Fig. S2], they were merged and defined as *KIT*^e8/WT^. As a result, *KIT*^D816/D820^ patients had significantly higher risk of relapse than both *KIT*^N822^ and *KIT*^e8/WT^ patients [59.8% (95% CI, 47.6–70.0%), 22.6% (95% CI, 4.3–49.4%) and 19.0% (95% CI, 8.8–32.2%), *P* = 0.0025 and <0.0001; censoring: *P* = 0.0009 and <0.0001, Fig. S2]. CIR was not significantly different between *KIT*^N822^ and *KIT*^e8/WT^ patients (*P* = 0.45, censoring: *P* = 0.19).

Next, patients were grouped according to MRD levels. The pretreatment baseline level of the *RUNX1-RUNX1T1* transcript was 388% in our center^4^. We selected the median value at CR and after 1st consolidation, 4.0% (2-log reduction compared to baseline) and 0.4% (3-log reduction) as the individual cutoff value. In agreement with our previous reports^4,10^, 0.4% was selected as the cutoff value for the timepoint of after 2nd consolidation. Thus, patients with *RUNX1-RUNX1T1* transcript levels higher and lower than the cutoff value were defined as high MRD levels and low MRD levels groups at individual timepoints. As shown in Fig. S3, patients with high MRD levels had significantly higher risk of relapse than those with low MRD levels at CR, after 1st consolidation and 2nd consolidation, respectively [CIR: 35.8% (95% CI, 24.2–47.6%) vs. 21.7% (95% CI, 9.6–36.9%), *P* = 0.0020; 38.1% (95% CI, 27.1–49.0%) vs. 17.4% (95% CI, 6.1–33.4%), *P* = 0.0001; 36.7% (95% CI, 23.7–49.8%) vs. 19.5% (95% CI, 8.4–34.0%), *P* = 0.0004].

Then *KIT* mutation status and MRD levels were combined, and patients who received allo-HSCT were censored at the time of transplantation. As shown in Fig. 1, for *KIT*^D816/D820^ patients, higher MRD levels at CR, after 1st consolidation and 2nd consolidation were significantly or tended to be significantly associated with an increased risk of relapse, respectively [74.2% (95% CI, 53.8–86.6%) vs. 55.9% (95% CI, 18.8–81.7%), *P* = 0.098; 81.8% (95% CI, 67.7–90.2%) vs. 40.0% (95% CI, 1.0–83.4%), *P* = 0.0048; 100.0% (95% CI, 100.0–100.0%) vs. 47.6% (95% CI, 9.7–79.0%), *P* = 0.0032]. Similarly for *KIT*^e8/WT^ patients, higher MRD levels at the three timepoints were significantly associated with an increased risk of relapse, respectively [43.4% (95% CI, 21.7–63.4%) vs. 14.3% (95% CI, 1.8–39.0%), *P* = 0.0008; 52.4% (95% CI, 29.7–70.9%) vs. 12.2% (95% CI, 1.4–35.7%), *P* < 0.0001; 56.5% (95% CI, 24.9–79.1%) vs. 17.0% (95% CI, 4.1–37.4%), *P* < 0.0001]. Whereas for *KIT*^N822^ patients, MRD levels at all three timepoints had no impact on relapse [CIR: 34.5% (95% CI, 3.1–72.0%) vs. 40.0% (95% CI, 2.4–79.8%), *P* = 0.80; 28.6% (95% CI, 0.7–73.3%) vs. 45.0% (95% CI, 6.5–79.2%), *P* = 0.50; 44.4% (95% CI, 2.7–83.3%) vs. 33.3% (95% CI, 3.2–70.3%), *P* = 0.81].

**Fig. 1:**
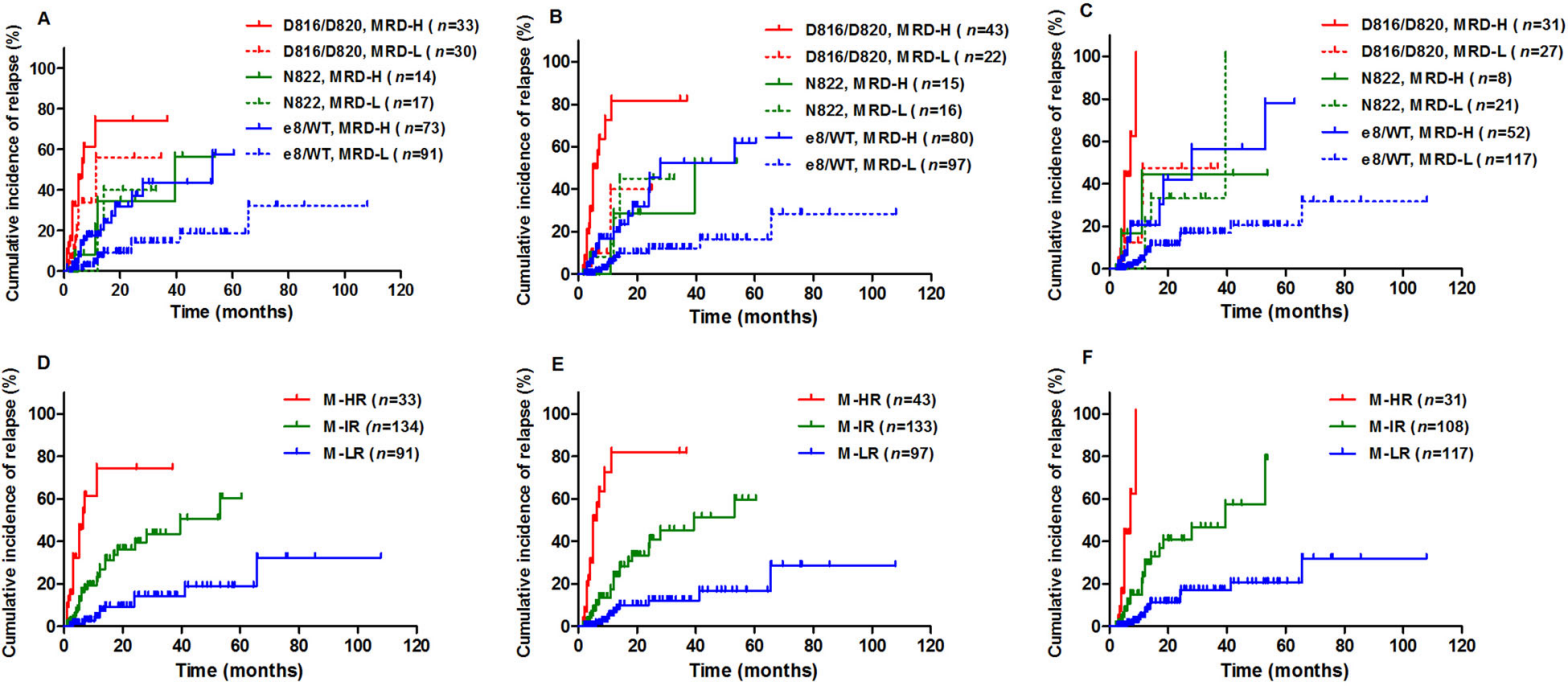
CIR with allo-HSCT patients censored at the time of transplantation and patients were categorized by the combination of *KIT* mutations and MRD status at different timepoints. **A**, **D** At CR; **B**, **E**: after 1st consolidation; **C**, **F**: after 2nd consolidation. MRD-H and MRD-L represented <2-log reduction and ≥2-log reduction of *RUNX1-RUNX1T1* at CR (**A**) and <3-log reduction and ≥3-log reduction of *RUNX1-RUNX1T1* after 1st and 2nd consolidation (**B**, **C**), respectively.

Furthermore, the four groups, *KIT*^D816/D820^ patients with low MRD levels, *KIT*^N822^ patients with high MRD levels, *KIT*^N822^ patients with low MRD levels and *KIT*^e8/WT^ patients with high MRD levels, had similar CIR at all three timepoints, respectively (*P* = 0.083, 0.94, and 0.94, Fig. 1A–C). Therefore, by considering *KIT* mutations and MRD status simultaneously, patients were recategorized into the following three groups: molecularly defined high-risk (M-HR; *KIT*^D816/D820^ patients with high MRD levels), molecularly defined intermediate-risk (M-IR; *KIT*^D816/D820^ patients with low MRD levels, *KIT*^N822^ patients, *KIT*^e8/WT^ patients with high MRD levels) and molecularly defined low-risk (M-LR; *KIT*^e8/WT^ patients with low MRD levels) groups. As a result, M-HR, M-IR and M-LR patients had significantly different 3-year CIR at CR, after 1st consolidation and 2nd consolidation, respectively [74.2% (95% CI, 53.8–86.6%) vs. 43.4% (95% CI, 26.9–58.8%) vs. 14.3% (95% CI, 1.8–39.0%), 81.8% (95% CI, 67.7–90.2%) vs. 45.3% (95% CI, 27.3–61.7%) vs. 12.2% (95% CI, 1.4–35.7%), 100.0% (95% CI, 100.0–100.0%) vs. 46.6% (95% CI, 26.6–64.4%) vs. 17.0% (95% CI, 4.1–37.4%); all *P* < 0.0001, Fig. 1D–F]. Therefore, MRD levels could identify patients with better prognosis from *KIT*^D816/D820^ and those with worse prognosis from *KIT*^e8/WT^ patients. It implied that *KIT* mutation and MRD levels had their unique prognostic roles and should be combined in order to better stratify t(8;21) AML.

Because t(8;21) AML patients are evaluated whether to recommend to receive allo-HSCT after 2nd consolidation in our center^4^, we just compared the outcomes between patients with different molecularly defined risk at the timepoint of after 2nd consolidation (*n* = 256). As shown in Fig. 2A, B, for M-HR patients (*n* = 31, 12.1%), allo-HSCT had both significantly lower CIR and significantly higher OS than chemotherapy alone [CIR: 38.4% (95% CI, 12.9–63.9%) vs. 100.0% (95% CI, 100.0–100.0%), *P* < 0.0001; OS: 76.9% (95% CI, 49.0–90.8%) vs. 0% (95% CI, 0–0%), *P* = 0.035]; for M-IR patients (*n* = 108, 42.2%), allo-HSCT had significantly lower CIR than and similar OS to chemotherapy alone [CIR: 13.2% (95% CI, 1.2–39.5%) vs. 53.2% (95% CI, 35.4–68.1%), *P* < 0.0001; OS: 92.2% (95% CI, 82.3–96.7%) vs. 76.8% (95% CI, 52.0–89.9%), *P* = 0.11]; for M-LR patients (*n* = 117, 45.7%), allo-HSCT had significantly lower CIR than chemotherapy alone [CIR: 0% (95% CI, 0–0%) vs. 18.2% (95% CI, 4.7–38.7%), *P* = 0.025], whereas, the OS was significantly lower for allo-HSCT than that for chemotherapy alone [78.7% (95% CI, 56.1–90.5%) vs. 95.6% (95% CI, 86.9–98.6%), *P* = 0.011]. Comparisons were further made within M-IR groups (Fig. 2C, D), and allo-HSCT had significantly or tended to have significantly lower CIR than and had similar OS to chemotherapy alone for all three groups, *KIT*^D816/D820^ patients with low MRD levels, *KIT*^N822^ patients and *KIT*^e8/WT^ patients with high MRD levels. (CIR: *P* = 0.094, 0.060 and <0.0001; OS: *P* = 0.13, 0.37 and 0.84).

**Fig. 2:**
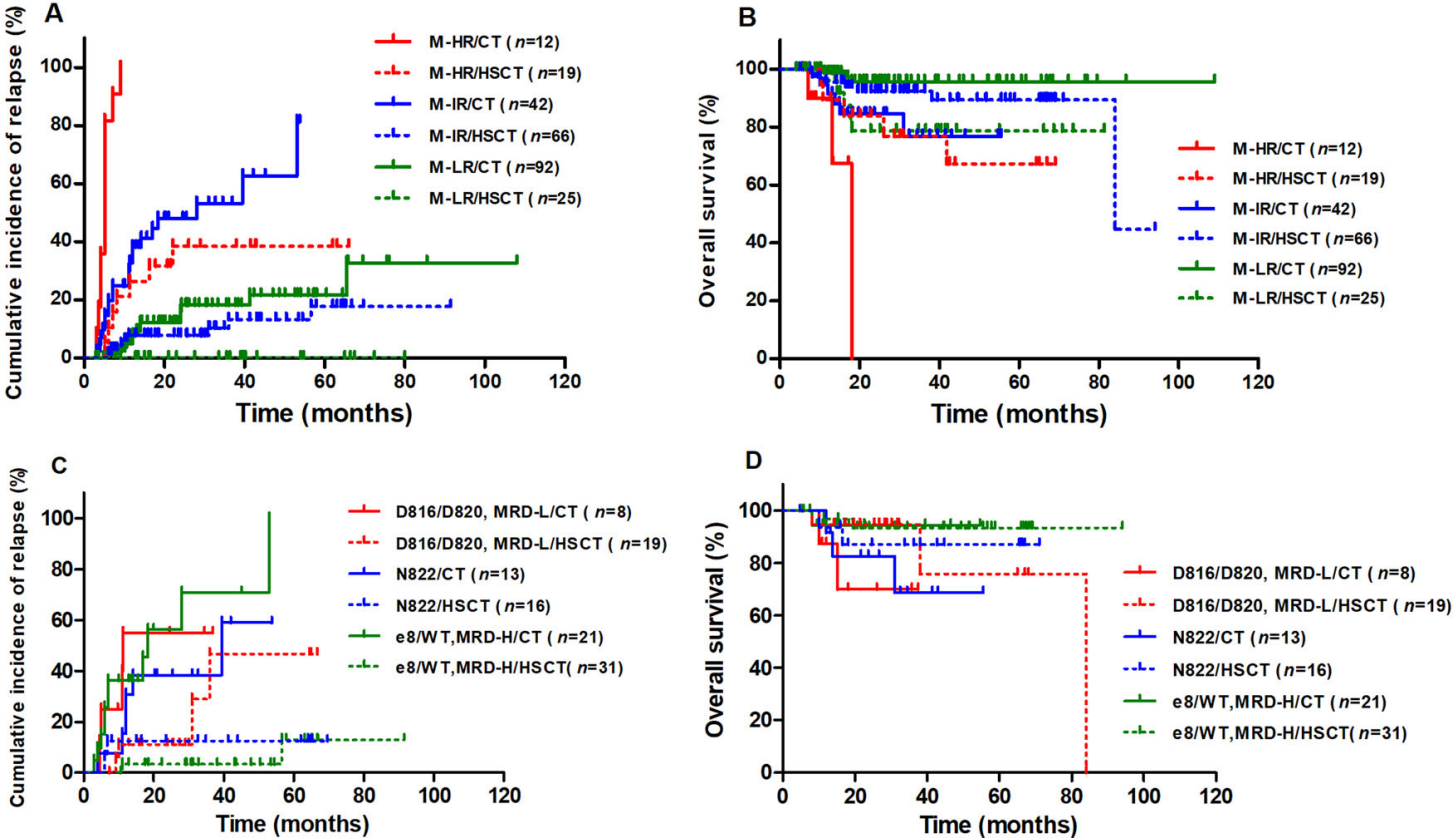
CIR and OS of patients categorized by the molecularly defined risk and treatment modality. **A**, **C**: CIR; **B**, **D**: OS. CT represented chemotherapy alone/auto-HSCT, HSCT represented allo-HSCT.

In summary, combination of *KIT* mutation and MRD levels improved risk stratification and treatment guidance in t(8;21) AML. *KIT*^D816/D820^ patients with <3-log reduction of *RUNX1-RUNX1T1* transcript levels after 2nd consolidation had the poorest prognosis and benefited from allo-HSCT on both relapse and survival; *KIT*^e8/WT^ patients with ≥3-log reduction after 2nd consolidation had the best prognosis, and allo-HSCT decreased not only relapse but also survival; the remaining patients had the intermediate prognosis and allo-HSCT decreased relapse but had no significant effect on survival. Multicenter prospective studies are warranted to confirm the current results.

## Supplementary information

Supplement

AJ-checklist
