# Supplementary material for: The impact of the combination of KIT mutation and minimal residual disease on outcome in t(8;21) acute myeloid leukemia
Source: Blood Cancer J. 2021 Apr 1;11(4):67. doi: 10.1038/s41408-021-00461-z (PMC8016839; doi:10.1038/s41408-021-00461-z)
Supplement: Supplementary file 1 — Supplement [file 41408_2021_461_MOESM1_ESM.pdf]

Supplement

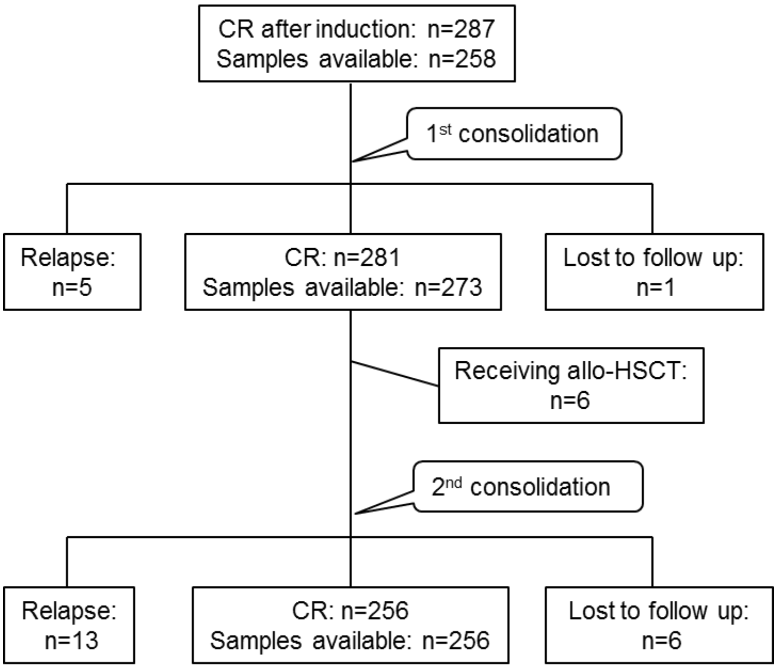

Figure S1. The flowchart of patient treatment and samples availability for the detection of *RUNX1-RUNX1* transcript before 2nd consolidation. 189 patients (65.9%) once included in our previous study which investigated the prognosis of the subtypes of *KIT* mutations (Reference 10) and their data were updated in the current study.

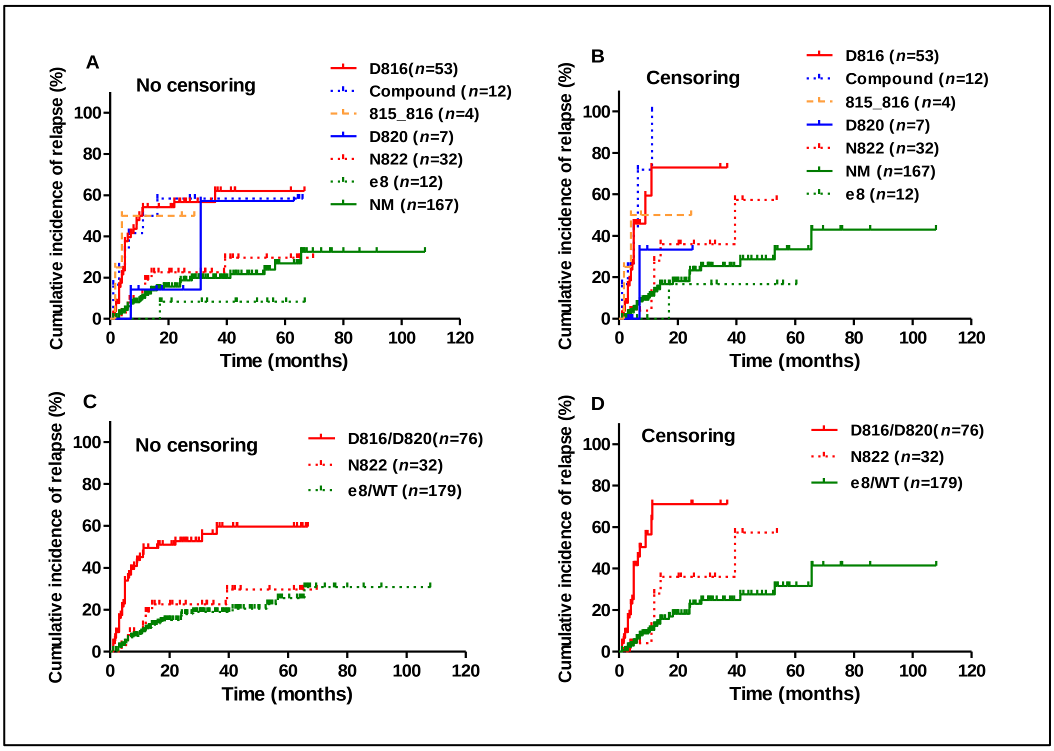

Figure S2. CIR of patients categorized by the *KIT* mutation status. A and C: all patients with no censoring; B and D: patients receiving allo-HSCT were censored at the time of receiving transplantation.

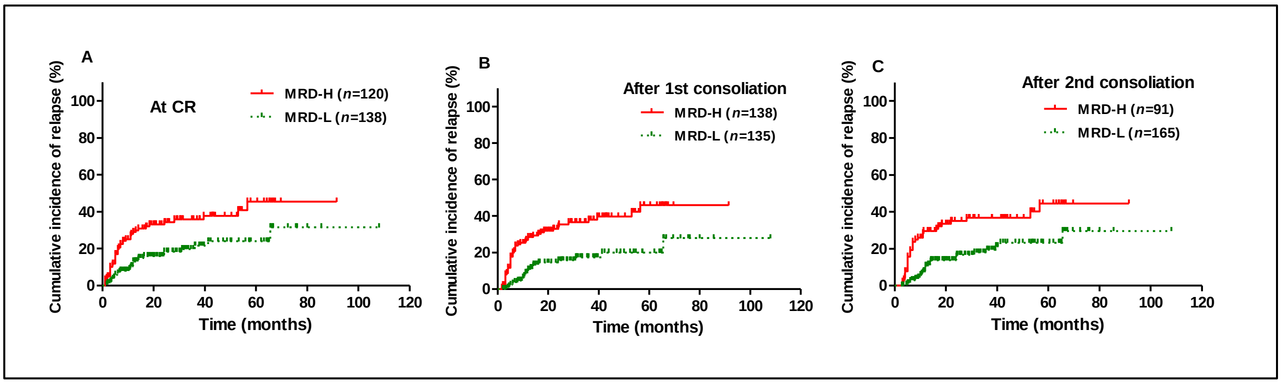

Figure S3. CIR of patients categorized by MRD status at different timepoints. A: at CR; B: after 1st consolidation; C: after 2nd consolidation. MRD-H and MRD-L represented <2-log reduction and ≥2-log reduction of RUNX1-RUNX1T1 at CR (A) and <3-log reduction and ≥3-log reduction of RUNX1-RUNX1T1 after 1st and 2nd consolidation (B and C), respectively.
